# The adverse impact of surveillance intervals on the sensitivity of FDG-PET/CT for the detection of distant metastases in head and neck cancer patients

**DOI:** 10.1007/s00405-016-4353-2

**Published:** 2016-11-01

**Authors:** Asaf Senft, Gül Yildirim, Otto S. Hoekstra, Jonas A. Castelijns, C. René Leemans, Remco de Bree

**Affiliations:** 1 Department of Otolaryngology-Head and Neck Surgery, VU University Medical Center, Amsterdam, The Netherlands; 2 Department of Radiology and Nuclear Medicine, VU University Medical Center, Amsterdam, The Netherlands; 3 Department of Head and Neck Surgical Oncology, UMC Utrecht Cancer Center, UMC Utrecht, Utrecht, The Netherlands

**Keywords:** Distant metastasis, Screening, FDG-PET/CT, Follow-up, Reference standard

## Abstract

The presence of distant metastases at initial evaluation influences treatment selection, since no effective systemic treatment for disseminated head and neck squamous cell carcinoma (HNSCC) is currently available. The reported sensitivity for the detection of distant metastases by contrast-enhanced (ce)CT and FDG-PET(/CT) differs substantially between studies. We hypothesized that these sensitivity values are highly dependent on the reference standard use, e.g., follow-up term. Therefore, we analyze our results of FDG-PET/CT (including chest ceCT) with long-term follow-up and compare these findings with data from the literature, with particular interest in the different reference standards. Forty-six HNSCC patients with high-risk factors underwent pretreatment screening for distant metastases by FDG-PET/CT (including chest ceCT). In 16 (35%) patients, distant metastases were detected during screening (6 patients) or during a mean follow-up of 39.4 months after screening (10 patients). The sensitivity and negative predictive value were 83.3 and 97.2% when 6 months, 60.0 and 89.9% when 12 months, and 37.5 and 72.2% when 30 months follow-up were used as reference standard, respectively. This is comparable with reported studies with similar reference standards. This critical appraisal on the reference standards used in our and reported studies shows room for improvement for the detection of distant metastases to refrain more patients from unnecessary extensive locoregional treatment for occult metastatic HNSCC.

## Introduction

Head and neck squamous cell carcinoma (HNSCC) accounts for approximately 5% of all malignant tumors worldwide. Two-thirds of the patients with HNSCC present with advanced disease. HNSCC preferentially metastasize to regional lymph nodes rather than spread hematogenously. Distant metastases usually occur late in the course of the disease. As results of locoregional treatment have improved significantly over the last decades, more patients are at risk to develop second primary tumors and distant metastases [1].

The presence of distant metastases at initial evaluation influences the prognosis and thus treatment selection: since no effective systemic treatment for disseminated HNSCC is currently available, patients with distant metastases are generally not considered curable and often receive only palliative treatment [2]. Therefore, screening for distant metastases is important to avoid futile treatments with extensive burden to patients and high costs.

The reported prevalence of clinically identified distant metastases in HNSCC at presentation is generally considered too low to warrant routine screening for distant metastases in all HNSCC patients. The risk of hematogeneous spread is directly related to the stage of disease, particularly to the presence and extent of lymph node metastases, and locoregional control. The yield of screening for distant metastases depends on the applied diagnostic methods [3]. High-risk factors have been identified and validated: ≥3 lymph node metastases, bilateral lymph node metastases, lymph node metastases ≥6 cm, low jugular lymph node metastases, regional recurrence, and second primary tumors [4–7]. Using these selection criteria, distant metastases were detected in 29–45% of the patients during initial screening using chest CT and/or FDG-PET (18–19%) or within 12-month follow-up (11–14%) [4–7]. Unfortunately, 20% of these high-risk patients who had a negative contrast enhanced CT (ceCT) of the chest at presentation developed distant metastases within 12 months after therapy with curative intent. In one-third of the cases, these missed distant metastases were extrathoracic.

We and others [8] have shown that adding FDG-PET to contrast-enhanced chest CT improves the accuracy and yield of staging, yielding a sensitivity of 63% with a horizon of 12-month follow-up in a prospective multi-center study [6]. However, still 15% of these high-risk patients who had a negative chest CT and whole body FDG-PET at presentation developed distant metastases within 12 months after therapy with curative intent [8]. Since in almost half of the patients the presence of distant metastases was missed, room for improvement remains.

New developments like the integrated combination of PET and CT (PET/CT) may improve the detection of (occult) distant metastases. A meta-analysis on integrated FDG-PET/CT showed for the detection of distant metastases and second primary cancers in head and neck cancer patients a pooled sensitivity of 89% and a specificity of 95% [9]. However, there was a striking range of sensitivity values (Table 1) [5, 6, 8, 12–18]. In previous studies with a long-term follow-up, we reported a sensitivity of only 55–63% [6, 19].We hypothesized that these sensitivity values are highly dependent on the reference standard use, e.g., follow-up term. Therefore, we analyze our results of FDG-PET/CT (including chest ceCT) with long-term follow-up and compare these findings with data from the literature, with particular interest in the different reference standards.

**Table 1:** Clinical studies on detection of distant metastases in HNSCC patients with follow-up as reference standard

Study	Technique	Patients		*N*	Sensitivity (%)	Specificity (%)	PPV (%)	NPV (%)	Follow-up (months)
Fakhry et al. [17]	CECT chest	All		37	100	92	86	100	6
Krabbe et al. [16]	CECT chest	All		82	55	63	21	88	>6
Brouwer et al. [5]	CECT chest	High risk		109	63	86	71	81	12
Brouwer et al. [5]	CECT chest	High risk	LRC	104	73	86	71	87	12
Senft et al. [6]	CECT chest	High risk		92	37 (24–52)	95 (88–98)	79 (57–91)	75 (66–82)	12
Senft et al. [6]	CECT chest	High risk	LRC	80	50 (33–67)	95 (88–98)	79 (57–91)	83 (75–90)	12
Ng et al. [8]^a^	CECT chest	All		160	50 (30–70)	98 (94–100)	81 (54–96)	91 (85–95)	12
Teknos et al. [13]	CECT chest	Advanced		12	33	100	100	33	24
Suenaga et al. [18]	CT chest	Recurrent^b^		170	33 (10–57)	99 (98–100)	83 (54–100)	94 (90–98)	>12
Krabbe et al. [16]	PET	All		149	85	94	58	98	>6
Senft et al. [6]	PET	High risk		92	53 (39–67)	93 (86–97)	80 (62–91)	80 (71–86)	12
Senft et al. [6]	PET	High risk	LRC	80	68 (51–82)	93 (86–97)	79 (61–90)	89 (80–94)	12
Ng et al. [8]^a^	PET	All		160	77 (56–91)	94.0 (89–97)	71 (51–87)	95.5 (90–98)	12
Teknos et al. [13]	PET	Advanced		12	100	100	100	100	24
Haerle et al. [12]	PET/non-ceCT	Advanced		299	97	95	67	100	6
Fahkry et al. [17]	PET/non-ceCT	All		37	92	85	73	96	6
Gourin et al. [14]	PET/non-ceCT	All		27	60	95	75	91	12
Gourin et al. [15]	PET/non-ceCT	Recurrent		64	86	84	60	95	
Suenaga et al. [18]	PET/non-ceCT	Recurrent^b^		170	53 (28–79)	99 (98–100)	89 (68–100)	96 (93–99)	>12
Haerle et al. [12]	PET/non-ceCT	Advanced		299	48	94	67	88	30@
Senft et al. [6]	PET + ceCT	High risk		92	63 (48–76)	95 (88–98)	86 (70–94)	84 (75–90)	12
Senft et al. [6]	PET + ceCT	High risk	LRC	80	82 (65–92)	95 (88–98)	86 (69–94)	93 (86–97)	12
Ng et al. [8]^a^	PET + ceCT	All		160	81 (61–93)	99 (95–100)	91 (72–100)	96 (91–99)	12
Suenaga et al. [18]	PET/ceCT	Recurrent^b^		170	60 (35–85)	99 (98–100)	90 (71–100)	96 (94–99)	>12

95% confidence intervals between brackets if available

*N* number of patients, *LRC* patients with locoregional recurrence and distant metastases during follow-up excluded

@median follow-up 30 months (range 1–72)

^a^Distant metastases and second primary tumors

^b^Suspicious for recurrence

## Materials and methods

### Patients and study design

We performed a retrospective cohort study on the yield of screening for distant metastases with whole body FDG-PET/CT (including chest ceCT) in high-risk head and neck cancer patients treated at the VU University Medical Center between April 2007 and August 2009. Patients were eligible for screening for distant metastases when meeting the following criteria: (1) HNSCC; (2) candidates for extensive treatment with curative intent (surgery and/or radiotherapy with or without chemotherapy); (3) minimum of 12-month follow-up; in case, no distant metastases were detected at initial presentation; (4) high-risk factors for development of distant metastases [7].

HNSCC was histologically confirmed in all cases, and all other histological subtypes were excluded. Because of their distinct metastatic patterns, squamous cell carcinoma of skin, nasopharynx, nasal cavity, and paranasal sinus was excluded. Finally, patients who rejected further workup, patients who died during the first year of follow-up due to other causes than metastatic HNSCC and those who were lost before 1-year follow-up because of other reasons were excluded.

Forty-six patients (33 men and 13 women) with a mean age of 61 years (range 33–76) met aforementioned criteria. These patients had the following high-risk factors: ≥3 lymph node metastases (*n* = 10), bilateral lymph node metastases (*n* = 13), lymph node metastases of ≥6 cm (*n* = 5), low jugular lymph node metastases (*n* = 5), regional recurrence (*n* = 7), and second primary tumours (*n* = 16), as assessed by palpation, CT, MRI, and/or ultrasound-guided fine-needle aspiration cytology. Some patients had more than one high-risk factor. Primary tumor sites were the oral cavity (*n* = 14), oropharynx (*n* = 16), hypopharynx (*n* = 8), and larynx (*n* = 11). Two patients had an unknown primary tumor. Five patients had synchronous second primary tumors. Patients were primary treated by surgery (*n* = 20), radiotherapy (*n* = 8), chemoradiation (*n* = 17), and chemotherapy (*n* = 1).

As part of the pretreatment workup, all patients underwent a panendoscopy, ce CT and/or magnetic resonance imaging (MRI) of the head and neck. If considered indicated, fine-needle aspiration of cervical lymph nodes was performed. Post-treatment follow-up was performed by regular visits to the outpatient clinic (every 6–8 weeks in the first year, with increasing intervals in following years). The mean follow-up was 39.4 months (range 1.7–90.2; median 30.2 months). No routine imaging screening for distant metastases was planned during follow-up, but additional examination was performed when suspicion arose either through the patient history or physical examination (e.g., weight loss, lesions/complaints suspicious of recurrence). Six patients developed a locoregional recurrence during follow-up.

### 18FDG-PET/CT

All patients underwent FDG-PET/CT pretreatment. During our study period, both the Gemini TF-64 and Ingenuity TF integrated PET/CT systems (Philips Medical Systems, Best, The Netherlands) were used to perform whole body (from mid-thighs to skull vertex) FDG-PET/CT scans, followed in the same scan session with ceCT of the chest. Patients fasted for at least 6 h prior to scanning, which started approximately 60 min after intravenous FDG administration. The dose administered was 2.5-MBq/kg body weight (±10%). Glucose levels were checked prior to 18FDG administration. Low-dose CT was performed with 120 kV and 50 mAs prior to emission scanning. PET–CT data were reconstructed using a time of flight row-action maximum likelihood algorithm, as implemented by the vendor. Final image matrix size equals 288 × 288 with a voxel size of 2 × 2 × 2 mm. Post-reconstruction image resolution was 5-mm full width at half maximum (FWHM).Preparation and scanning were performed according to EANM guidelines [10].

The FDG PET/low-dose CT images were interpreted by experienced nuclear medicine physicians and the ceCT scans by experienced radiologists, concluded with a joint reading session to integrate the findings. Readers had access to all relevant clinical information, according to common clinical practice. Most lesions suspicious of being malignant on FDG-PET/CT were confirmed using additional (follow-up) imaging, endoscopic workup and/or biopsy, using a rational approach. In a few cases, findings of FDG PET/CT were considered unequivocal regarding the presence of distant metastases, and consensus was reached not to perform additional workup by the multidisciplinary team.

### Scoring criteria

Radiological criteria for lung metastases were: (multiple) smoothly defined lesions mostly located subpleurally and at the end of a blood vessel. FDG uptake was considered suspicious for malignancy in the case of enhanced uptake incompatible with its physiological bio-distribution. In all patients, every scan report (chest ceCT and whole body FDG-PET/CT) was retrospectively scored for suspiciousness of distant metastases using a five-point ordinal Likert-scale: 0 = no lesion/uptake, 1 = definitively benign, 2 = probably benign, 3 = equivocal, 4 = probably malignant and 5 = definitely malignant. If more lesions were scored in a single patient, the lesion with the highest score was used for statistical analysis. The Likert scale was reduced to a binominal sensitive scale (0–2 = negative, 3–5 = positive) and conservative scale (0–3 = negative, 4–5 = positive) to obtain accuracy data for ceCT and FDG-PET/CT.

Criteria for combined and integrated chest ceCT and whole body FDG-PET/CT reading were based on a previous study [6]: positive if PET shows FDG uptake (Likert >0) or if PET shows no uptake and CT is positive (Likert 4 or 5) in small lesions below the detection limit (5 mm) of PET; and negative in all other scorings.

Although the primary goal was screening for distant metastases, we also registered second primary tumors. Patients with second primary tumors outside the head and neck region, which were found during screening, were described separately.

### Statistical analysis

FDG PET/CT or chest CT findings suspicious of being metastases were considered positive. If no suspicious lesion or lesions suspicious of being either benign or second primary tumors were found, the scan was considered negative. The FDG PET/CT and chest CT findings were compared to the findings of further initial workup and findings during follow-up. Negative findings on FDG PET/CT in patients who developed distant metastases during follow-up were considered as being false-negative, assuming these metastases were (subclinically) present at time of screening.

The result of the clinical diagnostic workup between screening until a follow-up of 12 months was used as reference standard, and patients were classified as positive or negative with respect to the presence of distant metastases. Other reference standards used were follow-up of 6 months and long-term follow-up.

In a separate analysis, these results were corrected for locoregional recurrence, since no distinction can be made between growth of subclinical metastases already present at the time of screening and reseeding of a locoregional recurrence after initial screening.

Sensitivity, specificity, positive, and negative predictive values of the chest ceCT, FDG PET/non-ceCT, and FDG PET/ceCT for detection of distant metastases were calculated.

## Results

In 22 of the total group of 46 patients (48%), distant metastasis (*n* = 16; 35%) or a second primary tumor (*n* = 6; 13%) was detected during screening or during follow-up after screening. Pretreatment screening identified distant metastases in six patients (13%) and a second primary tumor in 1 patient. Distant metastases were located in the lungs (*n* = 14), bone (*n* = 4), liver (*n* = 2), and skin (*n* = 1). In six patients, locoregional recurrence was observed; three of these patients developed distant metastases during follow-up.

Sensitivity, specificity, positive predictive value, and negative predictive value of the different imaging modalities, scoring, and reference standard are shown in Table 2. By sensitive reading and using a reference standard of 6 months, the sensitivity of ceCT, PET/non-ceCT, and PET/ceCT was 67.7, 66.7, and 83.3%, but these figures decreased when a follow-up of 30 months was used to 37.5, 25.0, and 37.5%, respectively.

**Table 2:** Results of scoring chest ceCT, whole body FDG-PET/CT and integrated PET/CT and ceCT using different reference standards (12 and 6 months and median 30.2-month follow-up) and conservative and sensitive reading and reading according to Senft et al. [6]

Scoring		Follow-up (months)	Sensitivity %	Specificity %	PPV %	NPV %
ceCT chest						
Conservative		30	18.8 (4.0–45.6)	96.7 (82.8–99.9)	75.0 (19.4–99.4)	69.0 (52.9–82.4)
		12	23.1 (5.0–53.8)	97.0 (84.2–99.9	75.0 (19.4–99.4)	76.2 (60.5–87.9)
		6	33.3 (7.5–70.1)	97.3 (85.8–99.9)	75.0 (19.4–99.4)	85.7 (71.5–94.6)
	LRC	30	23.1 (5.0–53.8)	96.7 (82.8–99.9)	75.0 (19.4–99.4)	74.4 (57.9–87.0)
		12	30.0 (6.7–65.2)	97.0 (84.2–99.9)	75.0 (19.4–99.4)	82.0 (66.5–92.5)
		6	67.7 (22.3–95.7)	97.3 (85.8–99.9)	80.0 (28.4–99.5)	94.7 (82.3–99.4)
Sensitive		30	37.5 (15.2–64.6)	83.3 (65.3–94.4)	54.5 (23.4–83.3)	71.4 (53.7–85.4)
		12	46.2 (19.2–74.9)	84.8 (68.1–94.9)	54.5 (23.4–83.3)	80.0 (63.1–91.6)
		6	67.7 (29.9–92.5)	86.5 (71.2–95.5)	54.5 (23.4–83.3)	91.4 (76.9–98.2)
	LRC	30	46.1 (19.2–74.9)	83.3 (65.3–94.4)	54.5 (23.4–83.3)	78.1 (60.0–90.7)
		12	60.0 (26.2–87.8)	84.8 (68.1–94.9)	54.4 (23.4–83.3)	87.5 (71.0–96.5)
		6	66.7 (22.3–95.7)	81.1 (64.8–92.0)	36.4 (10.9–69.2)	93.8 (79.2–99.2)
PET/non-ceCT						
Conservative		30	18.8 (4.0–45.6)	100.0 (88.4–100.0)	100.0 (29.2–100.0)	70.0 (53.9–82.8)
		12	30.0 (6.7–65.2)	100.0 (88.4–100.0)	100.0 (29.2–100.0)	83.7 (69.3–93.2)
		6	50.0 (11.8–88.2)	100.0 (88.4–100.0)	100.0 (29.2–100.0)	93.0 (80.9–98.5)
	LRC	30	23.1 (5.0–53.8)	100.0 (88.4–100.0)	100.0 (29.2–100.0)	75.0 (58.8–87.3)
		12	30.0 (6.7–65.2)	100.0 (88.4–100.0)	100.0 (29.2–100.0)	82.5 (67.2–92.7)
		6	50.0 (11.8–88.2)	100.0 (88.4–100.0)	100.0 (29.2–100.0)	92.5 (79.6–98.4)
Sensitive		30	25.0 (7.3–52.4)	90.0 (73.5–97.9)	57.1 (18.4–90.1)	69.2 (52.4–83.0)
		12	40.0 (12.2–73.8)	91.7 (77.5–98.2)	57.1 (18.4–90.1)	84.6 (69.5–94.1)
		6	66.7 (22.3–95.7)	92.5 (79.6–98.4)	57.1 (18.4–90.1)	94.9 (82.7–99.4)
	LRC	30	30.8 (9.1–61.4)	90.0 (73.5–97.9)	57.1 (18.4–90.1)	75.0 (57.8–87.9)
		12	40.0 (12.2–73.8)	90.9 (75.7–98.1)	57.1 (18.4–90.1)	83.3 (67.2–93.6)
		6	66.7 (22.3–95.7)	81.9 (78.1–98.3)	57.1 (18.4–90.1)	94.4 (81.3–99.3)
PET/CT and chest ceCT						
According to Senft et al. [6]		30	37.5 (15.2–64.6)	86.7 (69.3–96.2)	60.0 (26.2–87.8)	72.2 (54.8–85.8)
		12	60.0 (26.2–87.8)	89.9 (73.9–96.9)	60.0 (26.2–87.8)	89.9 (73.9–96.9)
		6	83.3 (35.9–99.6)	87.5 (73.2–95.8)	50.0 (18.7–81.3)	97.2 (85.5–99.9)
	LRC	30	46.1 (19.2–74.9)	86.7 (69.3–96.2)	60.0 (26.2–87.8)	78.8 (61.1–91.0)
		12	60.0 (26.2–87.8)	87.9 (71.8–96.6)	60.0 (26.2–87.8)	87.9 (71.8–96.6)
		6	60.0 (26.2–87.8)	87.9 (71.8–96.6)	60.0 (26.2–87.8)	87.9 (71.8–96.6)

*LRC* locoregional control (patients with locoregional recurrence and distant metastases during follow-up excluded), *PPV* positive predictive value, *NPV* negative predictive value

## Discussion

For the detection of distant metastases in HNSCC patients, chest CT and whole body FDG-PET are the most important diagnostic imaging techniques. However, studies are difficult to compare, and the real value is difficult to assess because of methodological differences. Unfortunately, some studies in head and neck cancer include tumor types other than HNSCC (e.g., nasopharyngeal carcinoma and salivary gland tumors) or sites with different clinical behavior (e.g., nasopharynx, nasal cavity, and paranasal sinus) and heterogeneous disease stages. The incidence of distant metastases (depending on type and stage) may influence predictive values of tests. Even more important is the reference standard used. Distant metastases that appear during follow-up in patients who achieved locoregional control must have arisen from subclinical distant spread already present at the time of treatment. Thus, if patients with locoregional disease control develop distant metastases despite negative screening, these distant metastases were missed (below the detection limit) by the technique used for screening. The best references are long-term follow-up and autopsy. The longer the follow-up, the higher the chance that occult distant metastases become manifest and the sensitivity and negative predictive value are expected to decrease. Spector et al. [11] performed a retrospective study on 170 patients who developed distant metastases: only 16.5% of patients had distant metastasis at presentation, and the remaining patients were diagnosed with distant metastases at a median of 324 days from HNSCC diagnosis [11]. In the study of Haerle et al. [12], the median time before metachronous (>6 months after screening) distant metastases became manifest was 11 months (range 7–34 months). Thus, only about half of the missed or metachronous distant metastases will be diagnosed within 12-month follow-up. In this study, the median follow-up was 30.2 months. The number of clinical studies with a clearly defined follow-up as reference standard is limited (Table 1).

Brouwer et al. [5] reported on 109 HNSCC patients with risk factors for distant metastases who underwent pretreatment screening by chest CT. Distant metastases were detected in 19% of these patients. Despite negative screening with chest CT, 9 (11%) patients developed distant metastases within a 12-month follow-up period. Using a follow-up of 12 months as reference standard and excluding patients with distant metastases as well as locoregional recurrence during follow-up, the sensitivity and specificity of the chest CT for the detection of distant metastases were 73 and 86%, respectively [5]. This is comparable with the sensitivity of 60% and specificity of 84.8% found in the present study. Using the same risk factors to select patients for screening also the predictive values are comparable. In a multi-center prospective study of Senft et al. [6], 92 patients with the same high-risk factors as used in this study (33% developed distant metastases), underwent screening for distant metastases by chest CT and whole body FDG-PET. Using a reference standard of 12-month follow-up, the sensitivity, specificity, positive predictive value, and negative predictive value of were for chest CT 37, 95, 79, and 75%, for FDG-PET 53, 93, 80, and 80% and for the combination (visual correlation) of chest and FDG-PET 63, 95, 86, and 84%, respectively. These figures improved when patients who developed distant metastases and locoregional recurrences simultaneously during follow-up were excluded, because no distinction can be made between growth of subclinical metastases already present at the time of screening and reseeding of a locoregional recurrence after initial screening: for chest CT 50, 95, 79, and 83%, for FDG-PET 68, 93, 79, and 89%, and for the combination (visual correlation) of chest and FDG-PET 82, 95, 86, and 93%, respectively [6].

Xu et al. [9] performed a meta-analysis on the accuracy of whole body FDG-PET/CT in staging of head and neck cancer. For the staging of head and neck cancer other than nasopharyngeal cancer, a pooled sensitivity and specificity of 88.8% [95% confidence interval (CI) 80.3–94.5] and 93.3% [95% CI 91.0–94.5%], respectively, were found for PET/CT. The pooled studies used different reference standards. The diagnostic value of PET/CT was not significantly better than PET only [9].

In 27 untreated HNSCC patients with mainly advanced HNSCC and 19% distant metastases, Gourin et al. [14] reported for the detection of distant metastases by FDG-PET/CT a sensitivity of 100%. However, when 12-month follow-up was used as reference standard, the sensitivity decreased to 60% and specificity, positive predictive value, and negative predictive value were 95, 75, and 91%, respectively [14]. In the later study of the same group [15] in 64 patients with suspected recurrent HNSCC following definitive treatment, the incidence of distant metastases was 23%. Using a reference standard of 12-month follow-up, the sensitivity, specificity, positive predictive value, and negative predictive value for the detection of distant metastases by PET/CT were 86, 84, 60, and 95%, respectively [15]. The higher sensitivity and lower specificity in this second group are suggestive for a more sensitive reading.

Krabbe et al. [16] reported on screening for distant metastases by FDG-PET in 149 HNSCC patients. In thirteen (8.7%) of these patients, distant metastases were detected during screening or follow-up of at least 6 months. Using this follow-up as the reference standard, a sensitivity of 85% and a specificity of 93% for FDG-PET were found. In the subgroup of 82 patients who underwent both FDG-PET and chest ceCT, these figures were 82 and 92% for FDG-PET, compared to 55 and 63%, respectively, for chest ceCT [16].

Ng et al. [8] compared the detection of distant malignancies (distant metastases and second primary tumors) by FDG-PET and extended-field ceCT of the chest in 160 newly diagnosed oropharyngeal and hypopharyngeal squamous cell carcinoma patients with negative results from chest radiography, liver ultrasound, and bone scanning, with a follow-up of 12 months. Twenty-six (16.3%) of these patients developed distant malignancies. The percentages of additionally detected distant malignancies by FDG-PET and ceCT were 12.5 and 8.1%, respectively. The sensitivity of FDG-PET was significantly higher (76.9 vs. 50.0%), while its specificity was slightly lower (94.0 vs. 97.8%) than ceCT. Visual correlation of FDG-PET and CT improved the sensitivity and specificity to 80.8 and 98.5%, respectively, leading to alteration of treatment in 13.1% of patients [8].

Haerle et al. [12] reported on 299 patients with advanced stage HNSCC who underwent screening for distant metastases using FDG-PET/non-ceCT. PET/CT detected distant metastases in 29 (10%) patients, while in 30 (11%) patients, distant metastases were diagnosed during a median follow-up of 30 months (range 1–72 months). A sensitivity of 97% and a specificity of 95% were reported using a reference standard of 6 months. When long-term follow-up was used as reference standard, the sensitivity decreased to 48% [12].

Recently, Suenaga et al. [18] reported on 170 patients previously treated for HNSCC with suspected recurrence who underwent PET/CT, consisting of non-ceCT and ceCT, to investigate. In 8.8% of the patients, distant metastases were detected during screening or follow-up of at least 12 months. The sensitivity and specificity for chest ceCT were 33 and 99%, for PET/CT with non-ceCT 53 and 99%, and for PET/CT with ceCT 60 and 99%, respectively. They concluded that the added value of ceCT at FDG-PET/CT is minimal, statistically not significant and likely not clinically relevant [18].

From the reported studies, it can be concluded that the specificity and negative predictive value for chest CT and whole body PET(/CT) are generally high. In the reported studies, when the follow-up (as reference standard) increased from 6, to 12, and to 24 months, the sensitivity for chest CT decreased from 100%, to 37–73%, and to 33%, respectively, and for the combination of PET and CT (visually correlated and integrated) from 92–97, to 63–82%, and to 48% (30 months). In this study, the accuracy was determined using the different reference standards in the same cohort of patients. The results of these analyses confirm the results found in the reported studies. This is illustrated by the sensitivity of the combination of chest ceCT and 18-FDG-PET/CT: 83% after 6-month follow-up, 60% after 12-month follow-up and 38% after a median follow-up of 30.2 months.

When only patients with locoregional control during surveillance were analyzed the sensitivity increased up to 23%. In this study, sensitive reading improved the sensitivity up to 34% for CT and up to 17% for PET/CT, while the specificity decreased but remained high.

Now, the question arises if for pretreatment screening for distant metastases these diagnostic techniques are sufficient enough? When it is the physicians’ opinion that an interval between HNSCC diagnosis and manifest distant metastases of at least 12 months justifies extensive locoregional treatment [20], one could argue that the sensitivity of 60–82% for the combination of PET and chest CT may be acceptable. Then, in the future studies, 12-month follow-up as reference standard will be sufficient.

Nevertheless, this critical appraisal on the reference standards used in the reported studies shows room for improvement for the detection of distant metastases. Due to the introduction of multi-channel receiver MRI, whole body MRI has become clinically feasible, with substantially reduced examination times [21]. Chan et al. [22] reported on 103 untreated oro- and hypopharyngeal carcinoma patients who underwent screening using FDG-PET–CT and WB-MRI. Distant metastases (*n* = 8) or second primary tumors (*n* = 10) were detected in 18 (17.5%) patients. Using a follow-up of at least 12 months as a reference standard, the sensitivity, specificity, positive, and negative predictive values of WB-MRI were 67, 96, 80, and 93%, respectively. The figures for PET–CT were 83, 95, 79, and 94%, respectively. For combined reading, these figures were 78, 98, 88, and 95%, respectively. The diagnostic capability of PET–CT seems higher, but this difference was statistically non-significant. Technical improvements like diffusion-weighted whole body imaging with background-body-signal-suppression (DWIBS) and experience in whole body MRI may increase the accuracy of this technique. With the rising concern of radiation dose in medical imaging, WB-MRI may be considered as a potential replacement for PET–CT for the whole body screening of patients. However, at the moment, none of these new methods have proved to be better for this topic. With the introduction of PET–MRI fusion studies, combined readings may improve the detection of distant metastases in the near future.

In conclusion, for pretreatment screening on distant metastases in HNSCC patients with high-risk factors, 18FDG-PET/ceCT should be performed. The reported accuracy, particularly sensitivity, of chest CT, 18FDG-PET/non-ceCT, and 18FDG-PET/ceCT for the detection of distant metastases is highly dependent on the reference standard used. A reference standard of 12 months may be sufficient, although still only half of the subclinical distant metastases missed during initial screening will become manifest within this time period. There is room for better diagnostic screenings techniques to refrain more patients from unnecessary extensive locoregional treatment for occult metastatic HNSCC.
